# Analysis of Related Risk Factors and Prognostic Factors of Gastric Cancer with Bone Metastasis: A SEER-Based Study

**DOI:** 10.1155/2022/3251051

**Published:** 2022-02-15

**Authors:** Chen Xiaobin, Xu Zhaojun, Liu Tao, Dai Tianzeng, Huang Xuemei, Zheng Fan, Huang Chunyin, Huang Jianqiang, Lin Chen

**Affiliations:** ^1^Department of General Surgery, 900th Hospital of Joint Logistics Support Force, Fuzhou 350001, China; ^2^Graduate School, Qinghai University, Xining 810001, China; ^3^Fuzong Clinical Medical College, Fujian Medical University, Fuzhou 350001, China; ^4^Dongfang Hospital, Xiamen University, Fuzhou 350001, China

## Abstract

**Background:**

Gastric cancer is among the most common malignant tumors at home and abroad, because its early symptoms are mostly insidious, which leads to distant metastasis when gastric cancer is first diagnosed. The common metastatic sites of gastric cancer are mainly the liver, lung, and peritoneum, but bone metastasis is relatively rare, and the prognosis of gastric cancer bone metastasis is very poor. Therefore, this study is built on the SEER database to analyze the related risk factors of gastric cancer bone metastasis and related factors affecting the prognosis of gastric cancer patients, aiming at improving clinicians' understanding of clinical diagnosis and prognosis of bone metastasis of gastric cancer, thus reducing misdiagnosis and missed diagnosis.

**Methods:**

The SEER database was collected to screen out patients with gastric cancer bone metastases and nonbone metastases matched with them from 2010 to 2016, and the Kaplan-Meier method was used to draw survival curves, and the comparison between survival curves was performed by Log-rank test to analyze the overall survival of the two groups of patient's time. Logistic regression analysis was used to analyze the related risk factors of gastric cancer bone metastasis, and the Cox regression proportional hazard model was used to analyze the relationship between gastric cancer bone metastasis and patient prognosis.

**Results:**

Using Kaplan-Meier survival curve to analyze the 1, 3, and 5-year survival rates of gastric cancer patients with bone metastasis and non-metastasis groups were 14.2%, 1.8%, 0.6% and 71.4%, 44.3%, 36.4%, respectively; the average survival rate of the metastatic group was The time was 4.0 months (95%CI: 3.475~4.525), and the average survival time of the non-metastatic group was 30.0 months (95%CI: 26.778~33.222). The difference between the two groups was statistically significant (*χ*^2^ = 1076.866, *P* < 0.001). Multivariate logistic regression analysis showed that race (*P* = 0.007, OR = 1.296), grade (*P* < 0.001, OR = 0.575), marital status (*P* < 0.001, OR = 0.040), tumor size (*P* = 0.006, OR = 0.752), TNM stage (*P* < 0.001), T stage (*P* = 0.023, OR = 0.882), and M stage (*P* < 0.001, OR = 44.958) are independent risk factors for gastric cancer bone metastasis. The Cox univariate analysis suggests that gastric cancer bone metastasis is a risk factor for the prognosis of gastric cancer patients. The Cox multivariate analysis validates that gastric cancer bone metastasis (HR = 0.584, 95% CI: 0.497~0.688, *P* < 0.001) is independent of the overall survival rate of gastric cancer patients.

**Conclusions:**

Race, grade, marital status, tumor size, TNM stage, T stage, and M stage are independent risk factors for gastric cancer bone metastasis; and gastric cancer bone metastasis is an independent risk factor that affects the prognosis of gastric cancer patients. Therefore, for such high-risk groups, large range screening of the above indicators can effectively improve the prognosis of gastric cancer patients to a certain extent.

## 1. Introduction

Gastric cancer is a common malignant tumor. According to the statistics of GLOBOCAN in 2020 [1], there are about 1.089 million new cases of gastric cancer worldwide and about 769,000 deaths. The incidence and mortality of malignant tumors which are, respectively, No. 5 and No. 4 seriously affect the quality of life and health of patients. Most patients with gastric cancer have insidious onset and no obvious symptoms in the early stage. In addition, people's lack of understanding of the disease results in the clinical diagnosis of gastric cancer, which is mostly advanced and metastasized. This brings great difficulties to the treatment of the disease, which leads to the risk of gastric cancer patients. The survival period is short. Therefore, understanding the status of gastric cancer metastasis will help reduce the damage to the body caused by metastasis and improve the prognosis of gastric cancer patients.

The common metastatic sites of gastric cancer are the liver, lung, and peritoneum, while bone metastasis is relatively rare. Although studies have reported that the autopsy of gastric cancer patients found that the bone metastasis rate reached 13.4%-15.9%, related studies reported that the probability of gastric cancer bone metastasis is relatively low, only 0.9%-2.1% [2–4]. Although the probability of gastric cancer bone metastasis is low, patients with gastric cancer bone metastasis are mostly accompanied by systemic multiple organ failure, which makes the patient's prognosis poor and greatly reduces the efficacy. On the other hand, the current clinical studies related to the analysis of risk factors and prognostic factors of gastric cancer bone metastasis are limited to small sample analysis or case reports, and further studies are needed to confirm or supplement the accuracy of the results. Therefore, this study is based on the SEER database to analyze and explore the risk factors of advanced gastric cancer bone metastasis and factors affecting the prognosis of gastric cancer patients, aiming to provide evidence for the diagnosis and treatment of advanced gastric cancer bone metastasis and improve the prognosis of patients.

## 2. Materials and Methods

### 2.1. Study Population

The SEER database [5] is the full name of the National Cancer Institute Surveillance, Epidemiology, and End Results Program (Surveillance, Epidemiology, and End Results Program), which records in detail the various types of patients in different states and counties in the United States since 1973 Information, including age, gender, race, year of diagnosis, marital status, insurance, tumor size, lymphatic metastasis (TNM) staging, and distant organ metastasis. The SEER database released information on bone metastases from gastric cancer in 2010. Therefore, this study used SEER∗Stat 8.3.6 software to extract the pathologically diagnosed cases of gastric cancer from 2010 to 2016. Inclusion criteria are as follows: (1) The primary tumor was located in the stomach when the patient was first diagnosed; (2) gastric cancer patients had only bone metastases; and (3) complete clinical, pathological, and follow-up data. Exclusion criteria are as follows: (1) Exclude secondary gastric cancer with bone metastasis; (2) gastric cancer without bone metastasis; (3) gastric cancer with liver or other sites metastasis; and (4) a large number of cases with incomplete data indicators.

### 2.2. Ethics and Consent

For the institutional cohorts, data were extracted from the Surveillance, Epidemiology, and End Results database. This article does not contain any studies with human participants performed by any of the authors. For this type of study, formal consent is not required.

### 2.3. The Indicators Included in the Analysis

The indicators included in the analysis are the following: age, gender, race, tumor site, histological type, grade, radiotherapy, chemotherapy, whether it is the primary lesion, insurance, marital status, tumor size, T stage, N stage, M stage, TNM stage, and other 16 factors after analysis.

### 2.4. Statistical Analysis

SPSS 26.0 and GraphPad Prism 8.0 were used to analyze the data, and the count data was expressed in the form of percentage *n* (%), and the comparison between groups was performed by the *χ*^2^ test. The Kaplan-Meier method was used to draw survival curves, and the comparison between survival curves was performed by Log-rank test to analyze the overall survival time of the two groups of patients. The logistic regression analysis was used to analyze the related risk factors of gastric cancer bone metastasis, and the Cox regression proportional hazard model was used to analyze the relationship between gastric cancer bone metastasis and patient prognosis. The difference was statistically significant with *P* < 0.05.

## 3. Results

### 3.1. General Data

A total of 2439 patients with gastric cancer were enrolled in this study, of which 951 had gastric cancer with bone metastasis and 1488 had no important organ (liver, brain, and lung) metastasis from gastric cancer, including 1584 (64.94%) males and 855 (35.06%) females, aged 17-103 (64.64 ± 13.651) years. Among them, 1666 cases (68.31%) died, and 773 cases (31.69%) were still alive.

### 3.2. Comparison of Pathological Characteristics between Patients with Gastric Cancer Bone Metastasis and Gastric Nonbone Metastasis

Bone metastasis of gastric cancer is related to age, race, tumor site, histological type, grade, insurance, marriage, tumor size, T stage, N stage, M stage, and TNM stage (*P* < 0.05) but has nothing to do with gender, radiotherapy, and chemotherapy (*P* > 0.05) (see Table 1).

### 3.3. Subsistence Analysis

**Figure 1:**
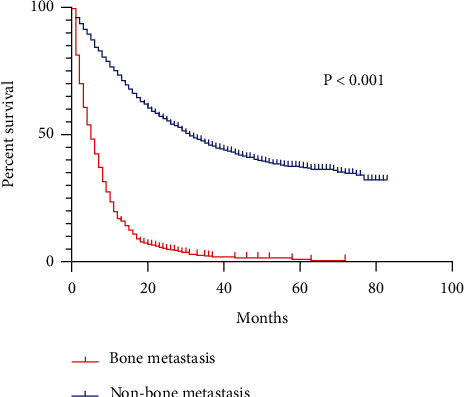
Overall 5-year survival rate of patients with gastric cancer bone metastasis and gastric cancer nonbone metastasis.

The average survival time of patients was 28.603 months (95% CI: 27.229~29.977), and the median follow-up time was 46 months (95% CI: 43.239~48.761). Among them, the median survival time of the bone metastasis group was 4.0 months (95% CI: 3.475~4.525) and the median survival time of the nonbone metastasis group was 30.0 months (95% CI: 26.778~33.222); patients with gastric cancer bone metastasis and nongastric cancer patients. The overall 1-year survival rates of patients with bone metastases were 14.2% and 71.4%, the 3-year survival rates were 1.8% and 44.3%, and the 5-year overall survival rates were 0.6% and 36.4%. The survival rate of the bone metastasis group was significantly lower than that of the nonmetastatic group. The overall survival rate difference between the two groups was statistically significant (*χ*^2^ = 1076.866, *P* < 0.05, Figure 1).

### 3.4. Logistic Regression Analysis of Single Factor Affecting Gastric Cancer Bone Metastasis

Univariate analysis results suggest that age, race, tumor site, grade, whether it is the primary tumor, insurance, marital status, tumor size, TNM stage, T stage, N stage, and M stage are related to gastric cancer bone metastasis (*P* < 0.05), and it has nothing to do with gender, histological type, radiotherapy, and chemotherapy (*P* > 0.05) (see Table 2).

### 3.5. Multivariate Logistic Regression Analysis of Influencing Gastric Cancer Bone Metastasis

The single-factor significant factors were included in the multivariate logistic regression equation. The results of stepwise regression analysis showed that race (*P* = 0.007, OR = 1.296), grade (*P* < 0.001, OR = 0.575), marital status (*P* < 0.001, OR = 0.040), tumor size (*P* = 0.006, OR = 0.752), TNM stage (*P* < 0.001), T stage (*P* = 0.023, OR = 0.882), and M stage (*P* < 0.001, OR = 44.958) are the factors affecting gastric cancer The independent risk factors of bone metastasis are shown in Table 3.

### 3.6. Evaluation of Diagnostic Efficacy Based on Risk Factors of Gastric Cancer Bone Metastasis

**Figure 2:**
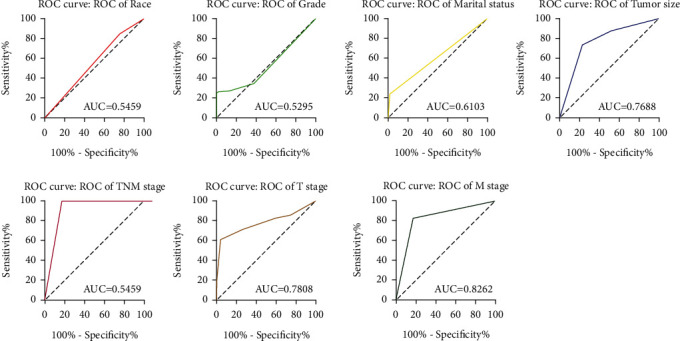
Race, grade, marital status, tumor size, TNM stage, T stage, and M stage to evaluate ROC curve of gastric cancer bone metastasis.

Through ROC curve analysis, we compared the correlation between independent risk factors and gastric cancer bone metastasis. The results showed that the AUC of race was 0.546 (95% CI: 0.523~0.569, *P* < 0.001); the AUC of grade was 0.529 (95% CI: 0.505~0.554, *P* < 0.001); the AUC of marital status was 0.610 (95% CI: 0.587~0.634, *P* < 0.001); the AUC of tumor size was 0.769 (95% CI: 0.749~0.788, *P* < 0.001); the AUC of TNM was 0.914 (95% CI: 0.902~0.926, *P* < 0.001); the AUC of T stage was 0.781 (95% CI: 0.760~0.802, *P* < 0.001); the AUC of M stage was 0.826 (95% CI: 0.808~0.844, *P* < 0.001), indicating that the tumor size is second only to the TMN staging and has a high correlation, as shown in Figure 2.

### 3.7. Analysis of Single Factor and Multiple Factors Affecting the Prognosis of Patients with Gastric Cancer

**Figure 3:**
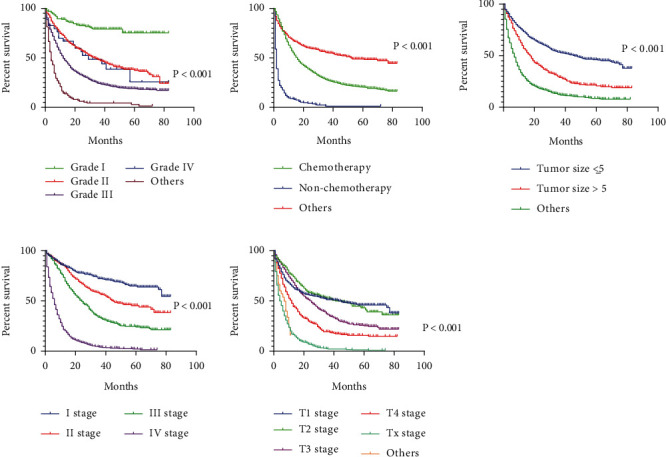
Survival analysis of independent risk factors affecting the prognosis of patients with gastric cancer.

The results of the Cox univariate analysis showed that marriage, race, gastric cancer metastasis, tumor site, histological type, grade, chemotherapy, tumor size, T stage, N stage, M stage, and TNM stage are the potential risk factors affecting the prognosis of gastric cancer patients. Significant factors were included in the Cox multivariate regression equation, and it was found that gastric cancer metastasis (HR = 0.548, 95CI: 0.487~0.688, *P* < 0.001), grade (HR = 0.960, 95% CI: 0.923~0.998, *P* = 0.041), chemotherapy (HR = 2.387, 95% CI: 2.142~2.660, *P* <0.001), tumor size (HR = 1.246, 95% CI: 1.157~1.341, *P* < 0.001), T stage, and TNM stage affect patients with gastric cancer independent risk factors for prognosis shown in Table 4 and Figure 3.

## 4. Discussion

In recent years, with the continuous improvement of the medical level, the detection rate of patients with potential bone metastases in the screening of gastric cancer patients has increased significantly. According to related research reports, the proportion of patients with suspicious gastric cancer bone metastases found in gastric cancer screening through bone scans is as high as 25%-45.3% [6, 7]. Therefore, further clinical research is needed to analyze the risk factors of gastric cancer patients with bone metastasis to improve the clinical diagnosis rate.

In this study, through univariate analysis of relevant clinicopathological factors affecting bone metastasis in patients with gastric cancer, it was found that age, race, tumor site, histological type, grade, insurance, marriage, tumor size, T stage, N stage, M stage, and TNM stage were related (*P* < 0.05) and have nothing to do with gender, radiotherapy, and chemotherapy (*P* > 0.05); in addition, through univariate and multivariate logistic stepwise regression analysis, the results suggest that race, grade, marriage, tumor size, T stage, M stage, and TNM stage are independent risk factors affecting bone metastasis of gastric cancer. Jingjing [8] analyzed the clinical data of 676 patients with gastric cancer in the First Affiliated Hospital of Zhengzhou University and found that the tumor TNM stage is an independent risk factor affecting gastric cancer bone metastasis, which is consistent with the results of this study. Min [9] also analyzed the risk factors related to gastric cancer bone metastasis and found that the degree of tumor differentiation was significantly related to gastric cancer bone metastasis, which was consistent with the results of this study. Bone metastasis mostly occurs in advanced gastric cancer, and the TNM stage of advanced gastric cancer is mostly at a higher value, and the degree of differentiation is lower than that in the early stage. These are the main reasons for gastric cancer bone metastasis.

In our study, survival analysis found that gastric cancer bone metastasis was significantly correlated with the overall prognosis of patients. The survival time of gastric cancer patients with bone metastasis was significantly shortened, and the 5-year survival rate was significantly lower than that of the gastric cancer nonbone metastasis group (0.6% vs 36.4%, *P* < 0.05); on the other hand, the results of this study showed that the median survival time of patients with gastric cancer with bone metastases was 4 months, which was consistent with the results of Lee's study [10]. The overall low survival rate of patients with gastric cancer bone metastasis is mainly due to the fact that it mostly occurs in advanced gastric cancer. On the one hand, advanced gastric cancer surgery is difficult and high risk; on the other hand, gastric cancer bone metastasis can cause disseminated intravascular coagulation (DIC), leading to patients' coagulation dysfunction to appear, followed by thrombosis, leading to complications such as bleeding and severe anemia. These are all contraindications to chemotherapy, and chemotherapy is the main treatment for advanced gastric cancer; therefore, the prognosis of patients with gastric cancer and bone metastasis is often compared to other types of gastric cancer. Patients with malignant tumors are poor [11]. For advanced gastric cancer, the diagnosis and treatment effect are not obvious. It is necessary to clarify the principle of its occurrence and use effective means to intervene to reduce the occurrence of bone metastasis in advanced gastric cancer. However, the molecular mechanisms involved in the occurrence of bone metastasis in gastric cancer are still unclear, mainly including the following points: ①At present, lymphatic and blood tract metastases are mostly considered, and blood tract metastasis is mainly considered. It is considered that cancer cells flow into the liver through the blood and then enter the right heart to participate in systemic circulation metastasis, while lymphatic metastasis mostly considered that cancer cells enter the systemic circulation through the thoracic duct to induce distant metastasis [12]; Chen et al. found that LNMAT1 can activate CCL2 by recruiting hnRNPL to the promoter region of CCL2 and stimulate VEGF through macrophages-C secretion to promote lymphatic metastasis, considering that gastric cancer may undergo lymphatic metastasis through this pathway [13]. ②The transcription factor Snail in gastric cancer cells inhibits the expression of miR-128 and activates the PI3K/AKP pathway to induce high expression of Bim-1, thereby promoting the tumor EMT process and assisting tumor cell metastasis [14]. ③The CpG islands in the promoter fragment of lncRNA SPRY4-IT1 induce the expression of SPRY4-IT1 by inhibiting the activity of DNA methyltransferase 1 (DNMT1), thereby promoting tumor metastasis. It is considered that gastric cancer may undergo bone metastasis through this pathway [15]. ④Mast cell protease antibody (MCPT) can stimulate the formation of tumor blood vessels, thereby providing nutritional support for bone metastasis of gastric cancer [16].

In this study, it was found that bone metastasis of gastric cancer is an independent risk factor that affects the prognosis of gastric cancer patients by studying related risk factors that affect the prognosis of gastric cancer patients. This result is consistent with the results of Lee et al. [10]. Nakanishi and others [17] pointed out that most patients with gastric cancer bone metastasis are accompanied by multiple sites, and most of the metastases are sites that are difficult to surgically remove. This is also the main reason for the poor prognosis of gastric cancer patients with bone metastases. In addition, the degree of differentiation, TNM stage, and tumor size are also independent risk factors that affect the prognosis of gastric cancer patients. The lower the degree of differentiation, the later the TNM stage, and the larger the tumor size, the poorer the prognosis of gastric cancer patients. Successively, Saito and Zhao also confirmed that the degree of tumor differentiation and tumor TNM staging is related to the prognosis of gastric cancer patients and can be used as an independent predictor of the prognosis of gastric cancer patients [18, 19]. The correlation between tumor size and the prognosis of gastric cancer is still controversial. Kooby et al. [20] found that tumor size is related to the prognosis of gastric cancer patients and is an independent risk factor affecting the prognosis of gastric cancer patients. Huang et al. [21, 22] believe that tumors are affected by many factors. The influence of factors, such as the operating skills of the surgeon, the depth of tumor invasion, and lymph node metastasis, cannot be used as factors to predict the prognosis of patients with gastric cancer.

This study has the following shortcomings: on the one hand, although the data sample size of this study is large, it still has selection bias as a retrospective study; on the other hand, some of the more important prognostic indicators are CEA, alkaline phosphatase, imaging diagnosis, etc. The data is not recorded in the SEER database in detail, and it is impossible to analyze whether the above factors have an impact on gastric cancer bone metastasis and whether there is an impact on the prognosis of gastric cancer patients. It is still necessary to increase the sample size and reference indicators to further verify its accuracy. In addition, this research shows that the tumor size is second only to the TMN staging and has a high correlation.

In summary, this article found through analysis, race, degree of differentiation, marriage, tumor size, TNM stage, T stage, and M stage are the independent risk factors affecting gastric cancer bone metastasis and gastric cancer metastasis is an independent risk factor affecting the prognosis of gastric cancer patients. Early detection of these risk factors is achieved by screening patients with gastric cancer. Diagnose and intervene as soon as possible in patients with gastric cancer bone metastases to effectively reduce the corresponding complications in patients with gastric cancer bone metastases and improve the prognosis of such patients.

## Figures and Tables

**Table 1:** Patient clinicopathological factors.

Characteristic	Bone metastasis	Nonbone metastasis	*χ* ^2^	*P* value
	*n* = 951	*n* = 1488		
Age at diagnosis			28.722	<0.001
≤60 y	412 (43.32%)	485 (30.78%)		
>60 y	539 (56.68%)	1003 (67.41%)		
Sex			1.619	0.203
Male	603 (63.41%)	981 (65.93%)		
Female	348 (36.59%)	507 (34.07%)		
Race			27.226	<0.001
White	707 (74.35%)	984 (66.13%)		
Black	94 (9.88%)	138 (9.27%)		
Others	150 (15.77%)	366 (24.60%)		
Tumor site			108.407	<0.001
Remote stomach	311 (32.70%)	700 (47.04%)		
Proximal stomach	404 (42.48%)	638 (42.88%)		
Whole stomach	236 (24.82%)	150 (10.08%)		
Histological type			73.077	<0.001
Adenocarcinoma	513 (53.94%)	946 (63.58%)		
Signet ring cell	327 (34.38%)	287 (19.29%)		
Others	111 (11.67%)	255 (17.14%)		
Grade			560.246	<0.001
Grade I	7 (0.74%)	160 (10.75%)		
Grade II	72 (7.57%)	375 (25.20%)		
Grade III	622 (65.40%)	925 (62.16%)		
Grade IV	11 (1.16%)	28 (1.88%)		
Unknown	239 (25.13%)	—		
Radiotherapy			0.048	0.816
Yes	297 (31.23%)	471 (31.65%)		
None	654 (68.77%)	1017 (68.35%)		
Chemotherapy			3.576	0.059
Yes	532 (55.94%)	890 (59.81%)		
None	419 (44.06%)	598 (40.19%)		
Primary			7.739	0.005
Yes	803 (84.44%)	1190 (79.97%)		
None	148 (15.56%)	298 (20.03%)		
Insurance			27.461	<0.001
Yes	888 (93.38%)	1453 (97.65%)		
None	63 (6.62%)	35 (2.35%)		
Marital status			302.502	<0.001
Married	724 (76.13%)	1461 (98.19%)		
Others	227 (23.87%)	27 (1.81%)		
Tumor size			168.201	<0.001
≤5 cm	116 (12.20%)	711 (47.78%)		
>5 cm	136 (14.30%)	435 (29.23%)		
Others	699 (73.50%)	342 (22.98%)		
T stage			994.704	<0.001
T1	137 (14.41%)	379 (25.47%)		
T2	30 (3.15%)	229 (15.29%)		
T3	108 (11.36%)	496 (33.33%)		
T4	97 (10.20%)	323 (21.71%)		
Tx	412 (43.32%)	61 (4.20%)		
Others	167 (17.56%)	—		
N stage			548.734	<0.001
N0	290 (30.49%)	697 (46.84%)		
N1	283 (29.76%)	392 (26.34%)		
N2	34 (3.58%)	148 (9.95%)		
N3	33 (3.47%)	222 (14.92%)		
Nx	144 (15.14%)	29 (1.95%)		
Unknown	167 (17.56%)	—		
M stage			1009.505	<0.001
M0	167 (17.56%)	1232 (82.80%)		
M1	784 (82.44%)	256 (17.20%)		
TNM stage			1588.475	<0.001
I	—	409 (27.49%)		
II	—	346 (23.25%)		
III	—	476 (31.99%)		
IV	951 (100%)	257 (17.27%)		

Note: Percentages may not total 100 because of rounding. Grade I: well differentiated; Grade II: moderately differentiated; Grade III: poorly differentiated; Grade IV: undifferentiated.

**Table 2:** Logistic regression analysis of single factor affecting gastric cancer bone metastasis.

Characteristic	*β*	OR (95% CI)	*P* value
Age at diagnosis	-0.458	0.633 (0.535~0.748)	<0.001
Race	-0.262	0.769 (0.694~0.853)	<0.001
Sex	0.110	1.117 (0.942~1.324)	0.203
Tumor site	0.579	1.785 (1.587~2.006)	<0.001
Histological type	0.076	1.079 (0.967~1.203)	0.175
Grade	0.380	1.463 (1.368~1.564)	<0.001
Radiotherapy	0.020	1.020 (0.856~1.215)	0.826
Chemotherapy	0.159	1.172 (0.994~1.382)	0.059
Primary	-0.307	0.736 (0.593~0.914)	0.006
Insurance	1.080	2.945 (1.932~4.490)	<0.001
Marital status	2.831	16.966 (11.273~25.534)	<0.001
Tumor size	1.346	3.843 (3.406~4.336)	<0.001
TNM stage	18.405	—	<0.001
T stage	0.761	2.141 (1.992~2.302)	<0.001
N stage	0.387	1.473 (1.389~1.562)	<0.001
M stage	3.118	22.593 (18.230~27.999)	<0.001

**Table 3:** Logistic regression analysis of multivariate factor affecting gastric cancer bone metastasis.

Characteristic	*β*	OR (95% CI)	*P* value
Age at diagnosis	0.009	1.009 (0.726~1.404)	0.956
Race	0.259	1.296 (1.073~1.564)	0.007
Tumor site	-0.13	0.878 (0.705~1.093)	0.245
Grade	-0.554	0.575 (0.487~0.679)	<0.001
Primary	-0.054	0.947 (0.611~1.468)	0.809
Insurance	-0.528	0.59 (0.259~1.345)	0.210
Marital status	-3.222	0.04 (0.013~0.127)	<0.001
Tumor size	-0.285	0.752 (0.613~0.922)	0.006
TNM stage	-22.031	2.70E-10	<0.001
T stage	-0.126	0.882 (0.791~0.983)	0.023
N stage	0.071	1.074 (0.959~1.203)	0.217
M stage	3.806	44.958 (6.068~333.109)	<0.001

**Table 4:** Cox univariate and multivariate analysis of gastric cancer metastasis.

Characteristic	Univariate analysis		Multivariate analysis	
	HR (95% CI)	*P* value	HR (95% CI)	*P* value
Age at diagnosis(years)		0.940		
≤60	Reference			
>60	0.996 (0.901~1.101)			
Sex		0.772		
Male	Reference			
Female	1.015 (0.918~1.123)			
Marital status		<0.001		
Married	Reference			
Others	2.494 (2.159~2.881)			
Race		0.002		
White	Reference			
Black	1.129 (0.961~1.326)			
Others	0.831 (0.735~0.940)			
Gastric cancer bone metastasis		<0.001		<0.001
Yes	Reference		Reference	
None	0.197 (0.177~0.220)		0.584 (0.497~0.688)	
Tumor site		<0.001		
Remote stomach	Reference			
Proximal stomach	1.165 (1.048~1.295)			
Whole stomach	1.655 (1.440~1.902)			
Histological type		<0.001		
Adenocarcinoma	Reference			
Signet ring cell	1.432 (1.284-1.597)			
Others	0.725 (0.622-0.845)			
Grade		<0.001	0.960 (0.923~0.998)	0.041
Grade I	Reference			
Grade II	0.530 (0.461~0.609)			
Grade III	0.171 (0.123~0.237)			
Grade IV	0.677 (0.448~1.023)			
Unknown	2.506 (2.158~2.910)			
Radiotherapy		0.369		
Yes	Reference			
None	1.048 (0.946~1.161)			
Chemotherapy		<0.001		<0.001
Yes	Reference		Reference	
None	1.255 (1.137~1.384)		2.387 (2.142~2.660)	
Whether it is a primary tumor		0.058		
Yes	Reference			
None	0.885 (0.779~1.004)			
Insurance		0.097		
Yes	Reference			
None	1.246 (0.961~1.616)			
Tumor size(cm)	1.909 (1.798~2.027)	<0.001	1.246 (1.157~1.341)	<0.001
≤5				
>5				
Others				
T stage		<0.001		0.011
T1	Reference		Reference	
T2	3.817 (2.976~4.902)		1.773 (0.240~13.115)	
T3	4.348 (3.289~5.747)		1.573 (0.212~11.686)	
T4	3.030 (2.387~3.861)		1.588 (0.215~11.719)	
Tx	1.873 (1.471~2.387)		2.085 (0.248~15.314)	
Others	1.234 (0.980~1.555)		1.900 (0.259~13.947)	
N stage		<0.001		
N0	Reference			
N1	3.049 (2.415~3.846)			
N2	1.825 (1.447~2.304)			
N3	2.604 (1.965~3.448)			
Nx	1.961 (1.517~2.532)			
Unknown	1.524 (1.175~1.977)			
M stage		<0.001		
M0	Reference			
M1	4.754 (4.281~5.281)			
TNM stage		<0.001		<0.001
I	Reference		Reference	
II	1.668 (1.321~2.108)		2.518 (1.918~3.305)	
III	2.880 (2.340~3.546)		3.999 (3.009~5.315)	
IV	10.335 (8.529~12.524)		10.878 (1.494~79.191)	

Note: Grade I: well differentiated; Grade II: moderately differentiated; Grade III: poorly differentiated; Grade IV: undifferentiated.

## Data Availability

The datasets generated and/or analyzed during the current study are available from the corresponding author on reasonable request.

## Additional Points

*Research Involving Human Participants and/or Animals*. This article does not contain any studies with human participants or animals performed by any of the authors.
